# RNAi Silencing of the Biomineralization Gene Perlucin Impairs Oyster Ability to Cope with Ocean Acidification

**DOI:** 10.3390/ijms24043661

**Published:** 2023-02-11

**Authors:** Caroline Schwaner, Emmanuelle Pales Espinosa, Bassem Allam

**Affiliations:** School of Marine and Atmospheric Sciences, Stony Brook University, Stony Brook, NY 11790, USA

**Keywords:** gene silencing, perlucin, oyster, bivalve, ocean acidification

## Abstract

Calcifying marine organisms, including the eastern oyster (*Crassostrea virginica*), are vulnerable to ocean acidification (OA) because it is more difficult to precipitate calcium carbonate (CaCO_3_). Previous investigations of the molecular mechanisms associated with resilience to OA in *C. virginica* demonstrated significant differences in single nucleotide polymorphism and gene expression profiles among oysters reared under ambient and OA conditions. Converged evidence generated by both of these approaches highlighted the role of genes related to biomineralization, including perlucins. Here, gene silencing via RNA interference (RNAi) was used to evaluate the protective role of a perlucin gene under OA stress. Larvae were exposed to short dicer-substrate small interfering RNA (DsiRNA-perlucin) to silence the target gene or to one of two control treatments (control DsiRNA or seawater) before cultivation under OA (pH ~7.3) or ambient (pH ~8.2) conditions. Two transfection experiments were performed in parallel, one during fertilization and one during early larval development (6 h post-fertilization), before larval viability, size, development, and shell mineralization were monitored. Silenced oysters under acidification stress were the smallest, had shell abnormalities, and had significantly reduced shell mineralization, thereby suggesting that perlucin significantly helps larvae mitigate the effects of OA.

## 1. Introduction

The eastern oyster (*Crassostrea virginica*) is an economically and ecologically important species that thrives along the Gulf and Atlantic coasts of the USA (full range from the Gulf of Mexico to Gulf of St. Lawrence and to the West Indies in the South [1]). Oysters provide vital services from controlling nutrient cycling and improving water quality to providing habitat for a diverse array of species [2]. In addition, oyster fishery and aquaculture support a multi-million-dollar industry [3]. Oyster reefs have been in decline due to overfishing and disease [4], and they now face the additional challenges related to climate change. The release of CO_2_ into the atmosphere from the combustion of fossil fuels has led to an unprecedented flux of excess CO_2_ into the ocean, resulting in alterations of carbonate chemistry and acidification of the seawater [5,6]. The shell of the eastern oyster is composed of 95% calcium carbonate (CaCO_3_) [1], which makes oysters vulnerable to ocean acidification (OA) because it is more difficult to precipitate CaCO_3_ under low-pH conditions. The eastern oyster is found in estuarine environments with variable pH and extreme acidification events [7,8] and must have at least some capacity to tolerate alterations in the carbonate chemistry of seawater. Previous research has demonstrated that organisms are able to respond to OA through various means including physiological mechanisms [9,10,11], gene expression [12,13,14], or local adaptations [15,16,17]. However, what makes certain species, populations, and individuals resilient to OA is still not fully understood.

There have been multiple investigations to attempt to identify processes conferring resilience, but these are often large-scale ‘omic’ investigations that culminate in hundreds of potential variants, genes, or proteins. While there is much variability, commonalities such as particular groups of genes or pathways have emerged. One group of genes, perlucins, has cropped up over and over in the literature and from previous in-house studies of the eastern oyster. For example, perlucin genes have been shown to be upregulated under OA stress in the Manila clam [18], sea snails [19,20], Sydney rock oyster [13], Mediterranean mussel [21], and Mexican geoduck [22]. Our own previous work showed that oysters (*C. virginica*) that survived selective mortality from OA conditions had significant enrichment of a missense single nucleotide polymorphism (SNP) in a perlucin gene. In the same group of oysters, perlucins were also significantly upregulated under OA stress at both larval and juvenile stages. Perlucin genes were also upregulated in different cohorts and populations of oysters as well as different life stages and populations of hard clams (*Mercenaria mercenaria*) under OA conditions [23,24].

There are over 100 perlucin genes in the *C. virginia* genome [25], and many are without verification of their function. Perlucin is a typical C-type lectin which contains a carbohydrate-binding feature that facilitates calcium-dependent glycoprotein interactions with the skeletal matrix [26]; however, the specificity of binding remains unknown. Studies in other species have suggested that perlucins have a function in calcification, specifically the crystallization of CaCO_3_ [27,28,29]. In fact, perlucin was first isolated from the nacre of the abalone inner shell, and it was found that perlucin increased the precipitation of CaCO_3_ from a saturated solution [27] and nucleated and modified the morphology of CaCO_3_ crystals [28]. We have hypothesized that perlucin is upregulated under OA conditions because it helps to sustain calcification, which is adversely impacted by low pH.

To test this hypothesis, we used RNA interference (RNAi), which is a conserved pathway that can be utilized by researchers to “silence a gene” for functional analysis [30,31]. Briefly, it is a pathway of proteins that cleaves double-stranded RNA (dsRNA) in a sequence-specific manner that results in downregulating or silencing a gene [32]. While most RNAi studies of invertebrates have used insects, crustaceans, and nematodes [32], this method has also been effective in bivalves [33,34,35,36,37,38]. However, most information has been on adult bivalves, with injection of dicer-substrate small interfering RNA (DsiRNA) into individual adductor muscles [34,36], which is not possible in larvae. We were interested in developing RNAi for bivalve larvae. Previous studies explored the application of RNAi in copepods using different methods including feeding, lipofection, rehydration, and electroporation [39]. Feeding and rehydration showed no evidence of knock-down. Lipofection had few instances of gene knock down, but the suppression levels were inconsistent, weak, and associated with high mortality. Similarly, electroporation caused high mortality in the copepods [39]. Eichner et al. (2014) used a previously described protocol for gene silencing in planktonic salmon lice stages; however, they found no reproducible downregulation of target genes [40]. RNAi was also attempted in rotifers using soaking, ingestion, electroporation, and microinjection with a variety of chemical transfection agents [41]. Results of these trials were highly variable and successful transfections required the decapsulation of eggs and the use of lipofectant [41]. RNAi has also been tried and failed in many animals and the exact reasons for the variability of outcomes are unknown [42]. Nevertheless, a simple soaking method was successful in nematode eggs [43] and freshly hatched nematodes [44].

There are only a few papers on RNAi in bivalve larvae. Yang et al. (2017) soaked oyster (*Crassostrea angulata*) trochophore larvae in dsRNA, but the authors did not report if the method caused mortality and there was no information on larva stocking densities, which are known to affect survivorship in bivalves [35,45,46]. Liang et al. (2019) performed RNAi on the pediveliger stage of mussels using electroporation [37]. Similarly, Wang et al. (2022) utilized electroporation on dwarf surfclam eggs, but they did not report mortality levels induced by the transfection method [38]. In fact, several previous RNAi studies reported a high level of mortality when electroporation was used [39,47,48].

Here, the objective was to test the hypothesis that perlucin plays a role in resilience to OA in the eastern oyster using a gene interference technique. We hypothesized that high expression of perlucin helps oysters sustain calcification under acidified conditions and maintains the structural integrity of larval shells, whereas without perlucin, larval shell calcification will be compromised and have irregular development. Because of the limited information on RNAi in bivalve larvae and the variability and irreproducibility reported in other invertebrates, two different transfection approaches were implemented to optimize the chances of success. Results highlight the importance of perlucin in protecting oyster larvae from the negative effects of OA.

## 2. Results

### 2.1. Perlucin Expression and Transfection Success

**Figure 1:**
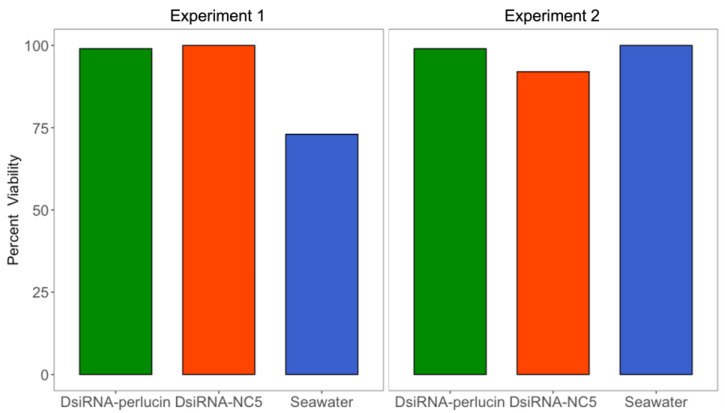
Viability of larvae post-transfection (after 4 h for Experiment 1 and 24 h for Experiment 2) and prior to exposure to *p*CO_2_ treatments was assessed. Transfection of DsiRNAs (gene silencing (green) and negative control (orange)) did not induce mortality and larval viability was similar to those without transfection (blue). There are no error bars or statistics performed because all replicates were pooled for the transfection step.

Two transfection approaches were evaluated. The first method (Experiment 1) involved the exposure to DsiRNA during the fertilization process, while the second method (Experiment 2) relied on the exposure of developing larvae to DsiRNA. First, we evaluated the effect of transfection on larvae viability before exposure to *p*CO_2_ conditions (4 h and 24 h post-transfection for Experiment 1 and Experiment 2, respectively) to ensure that the transfection methods themselves did not induce mortality. Results showed high survivorship (99%) in larvae transfected with DsiRNA-perlucin using either method (Figure 1). Survivorship of larvae that received the control probe (DsiRNA-NC5) was also high (100% and 92% for Experiments 1 and 2, respectively; Figure 1), while the viability among control larvae (seawater treatment) was 73% for Experiment 1 and 100% for Experiment 2.

**Figure 2:**
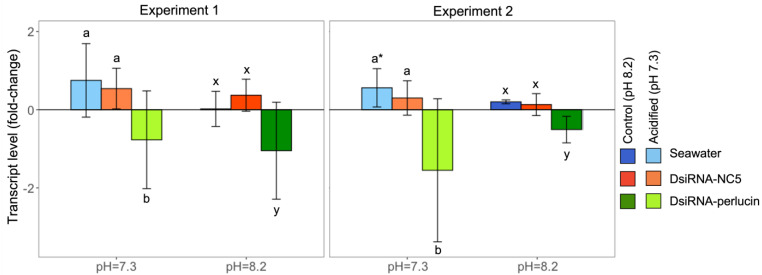
Relative change in perlucin expression (mean ± sd) among silenced oysters (DsiRNA-perlucin (green)) compared to controls (DsiRNA-NC5 (orange) and seawater (blue)) grown at pH = 7.3 (lighter shades) or pH = 8.2 (darker shades). Different letters (a and b for pH 7.3 and x and y for pH 8.2) indicate significant differences within each pH group (1-way ANOVA followed by Student–Newman–Keuls post hoc tests, *n* = 3 pools of larvae per data point). The * denotes significant over-expression of perlucin among non-silenced oysters (treated with seawater) grown under acidified conditions (pH = 7.3) as compared to those cultured at pH = 8.2 (*t*-test, *p* < 0.05). Oysters were sampled on day 3 for Experiment 1 and day 5 for Experiment 2.

Before looking at the consequences of gene silencing on larvae resilience, or lack thereof, to low pH, it was necessary to (1) confirm the upregulation of perlucin in non-silenced oysters under OA and (2) assess the effect of the transfection methods on gene expression. As expected, perlucin expression was higher in larvae grown under acidified conditions as compared to control conditions (Figure 2). However, this upregulation was statistically significant for Experiment 2 (day 5, *t*-test, *p* = 0.003) but not for the first experiment (day 3).

The second step was to confirm if RNAi was successful and evaluate the two methods for decreasing the expression level of perlucin in experimental groups that received DsiRNA. Results showed that both transfection methods successfully reduced the expression of the target perlucin in silenced oysters as compared to DsiRNA-NC5 or seawater controls (Figure 2, Table S1). In addition, it is important to note that no differences in perlucin expression were observed between controls (i.e., DsiRNA-NC5 and seawater), suggesting no effect linked to the introduction of non-target DsiRNA.

Overall, both methods successfully reduced the expression of perlucin and did not cause high mortality; however, some differences between the two approaches were identified and are discussed below.

### 2.2. Viability

**Figure 3:**
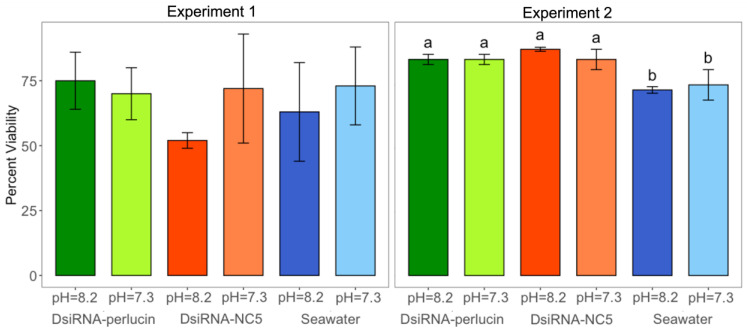
Viability (mean ± sd) of silenced (DsiRNA-perlucin (green)) and control (DsiRNA-NC5 (orange) and seawater (blue)) oyster larvae cultured under different *p*CO_2_ treatments (lighter shades for pH = 7.3 and darker for pH = 8.2). Different letters (a and b) denote significant differences (1-way ANOVA, *n* = 3, *p*-value < 0.05) in Experiment 2 (no significant differences were identified for Experiment 1). Larvae were sampled on day 3 for Experiment 1 and day 5 for Experiment 2.

The first assay to determine differences in susceptibility to high-*p*CO_2_ conditions among control and silenced oysters was viability. Silenced oysters had similar mortality levels as their respective controls. In fact, there were no significant differences in viability of oyster larvae between any treatments for Experiment 1 (larvae collected 3 days post-transfection, 1-way ANOVA; *p*-value = 0.4255; Figure 3). For Experiment 2, larvae with seawater only (no DsiRNA) in both pH = 7.3 and pH = 8.2 were less viable than larvae in all other treatments (5 days post-transfection, 1-way ANOVA; *p*-value <0.001; Figure 3).

### 2.3. Shell Development: Length and Deformities

**Figure 4:**
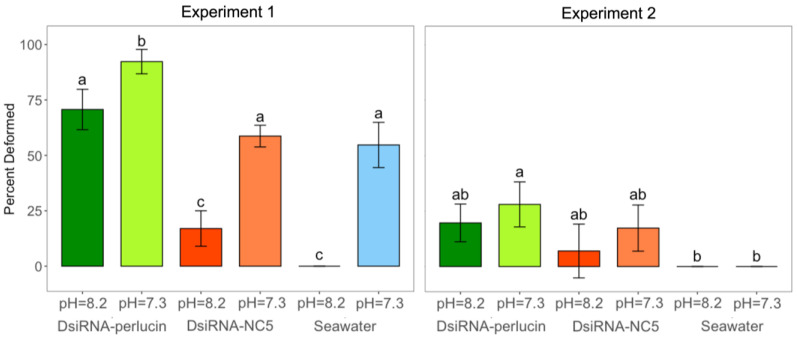
Percentage of deformed larvae (mean ± sd) among silenced (DsiRNA-perlucin (green)) and control (DsiRNA-NC5 (orange) and seawater (blue)) oysters cultured under different *p*CO_2_ treatments (lighter shades for pH = 7.3 and darker for pH = 8.2). Different letters (a, b, and c in Experiment 1 and a and b in Experiment 2) denote significant differences between treatments within each experiment (1-way ANOVA, Tukey post hoc tests; *p*-value < 0.05, *n* = 3). Larvae were sampled on day 3 for Experiment 1 and day 5 for Experiment 2.

**Figure 5:**
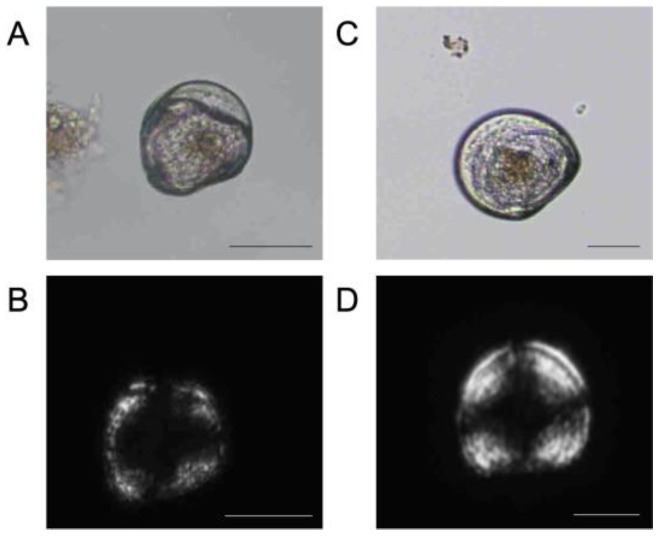
Photomicrographs showing altered (**A**,**B**), from the DsiRNA-perlucin/pH = 7.3 treatment) and normal (**C**,**D**), from control no DsiRNA treatment reared at pH = 8.2) larvae viewed under brightfield (**A**,**C**)) or cross-polarized light (**B**,**D**) microscopy. Note the lower refringence in B indicative of lower biomineralization as compared to (**D**). Images taken with Nikon Eclipse TE2000-S inverted microscope. Scale bars = 50 μm.

While silenced oysters did not have greater mortality, they did have dramatic differences in development in Experiment 1, where silenced larvae grown in acidified seawater had a significantly greater percentage of larvae with shell deformities (92%) as compared to the DsiRNA-NC5 (59%) and seawater (55%) controls grown at the same pH (day 3; 1-way ANOVA with Tukey post hoc tests, *n* = 3, *p*-value < 0.001; Figure 4). In the pH = 8.2 treatment, the DsiRNA-perlucin treatment induced a significantly greater percentage of deformed larvae (71%) as compared to DsiRNA-NC5 (17%) and seawater only (0%). The example of deformed larvae versus normal larvae is shown in Figure 5.

**Figure 6:**
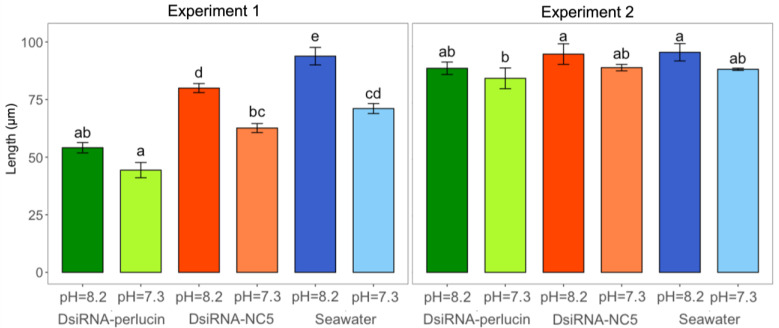
Length (mean ± sd) among silenced (DsiRNA-perlucin (green)) and control (DsiRNA-NC5 (orange) and seawater (blue)) oysters cultured under different *p*CO_2_ treatments (lighter shades for pH = 7.3 and darker for pH = 8.2). Different letters (a, b, c, d, and e in Experiment 1 and a and b in Experiment 2) denote significant differences between treatments within each experiment (nested ANOVA; Tukey post hoc test, *n* = 3; *p*-value < 0.04). Silenced larvae were systematically smaller than their respective controls (DsiRNA-NC5 and seawater) and differences were significant for Experiment 1. Larvae were sampled on day 3 for Experiment 1 and day 5 for Experiment 2.

Larvae from Experiment 1 had significant differences in growth as well (day 3; nested ANOVA, Tukey post hoc; *n* = 3, *p*-value < 0.0001; Figure 6). The smallest animals were from the DsiRNA-perlucin/pH = 7.3 and DsiRNA-perlucin/pH = 8.2 treatments. Silenced larvae in acidified seawater (44 ± 2 µm) were significantly smaller than all other treatments except the DsiRNA-perlucin pH = 8.2 treatment (54 ± 4 µm). Within acidified conditions, silenced larvae were significantly smaller than larvae with DsiRNA-NC5 (63 ± 5 µm) and seawater (71 ± 2 µm) and both negative controls were not significantly different from each other. Within ambient conditions, larvae with DsiRNA-NC5 (80 ± 3 µm) and seawater (94 ± 1µm) were significantly larger than silenced larvae (54 ± 4 µm).

Results from Experiment 2 (samples 5 days post-transfection) followed the same general trends as those from the first experiment, with greater percentages of deformities among silenced larvae (1-way ANOVA, *n* = 3, *p*-value < 0.05; Figure 5). Silenced larvae grown in acidified conditions had a significantly greater percentage of larvae (28%) with shell deformities than did non-silenced larvae (treated with seawater) grown in acidified seawater (0%). At day five, silenced larvae grown in acidified seawater were smaller (86 ± 5 µm) than larvae from seawater (90 ± 0.5 µm) and DsiRNA-NC5 (91 ± 1 µm) controls reared at the same pH, although differences were not significant (Figure 6). They were however significantly smaller than control larvae reared at pH 8.2 (97 ± 4 µm for seawater only and 97 ± 5 µm for DsiRNA-NC5).

### 2.4. Biomineralization

As stated in the “Materials and methods” section, the number of larvae used in Experiment 1 was low and only permitted the collection of one replicate for biomineralization (Figure S1). We were unable to perform statistical analysis due to the lack of replication.

**Figure 7:**
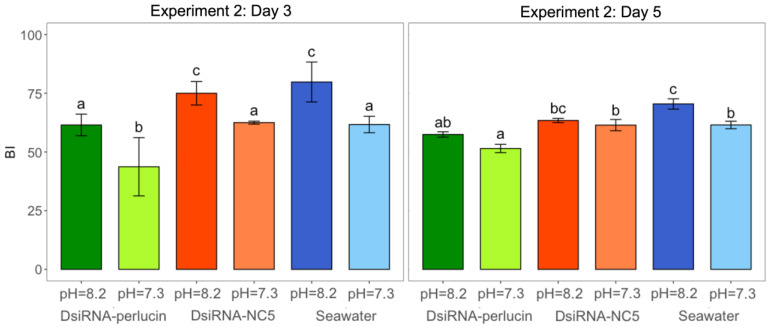
Shell birefringence index (BI, mean ± sd) among silenced (DsiRNA-perlucin (green)) and control (DsiRNA-NC5 (orange) and seawater (blue)) oysters cultured under different *p*CO_2_ treatments (lighter shades for pH = 7.3 and darker for pH = 8.2) of oyster larvae from Experiment 2. BI was used as a proxy to measure biomineralization under cross-polarized light microscopy. Different letters (a, b, and c in each of Experiments 1 and 2) denote significant differences between treatments within each sampling day (nested ANOVA, *n* = 3, *p*-value < 0.05; Tukey post hoc tests).

Larvae from Experiment 2 had significant differences in biomineralization (Figure 7) between treatments for both sampling points. For the first sampling point (day 3), silenced larvae in acidified and control conditions had significantly less biomineralization than their respective controls (nested ANOVA; *n* = 3; *p*-value < 0.05; Tukey post hoc). Apart from silenced larvae in pH = 8.2, all larvae in acidified conditions had significantly less biomineralization than larvae reared at pH = 8.2. At the second sampling point (day 5), silenced larvae in OA conditions had significantly less biomineralization than the OA controls; however, they had similar biomineralization as silenced larvae in pH = 8.2. For both days and both *p*CO_2_ conditions, the DsiRNA-NC5 and seawater treatments did not have any effect on mineralization.

## 3. Discussion

This study demonstrates that perlucin helps mitigate the effects of OA in larval eastern oysters and highlights the role of this perlucin gene in larval shell formation. Two RNAi transfection methods were used to increase the chance of successfully silencing perlucin, and both methods were successful. It was hypothesized that by reducing the expression of perlucin, there would be a significant decrease in viability, shell biomineralization, and irregular development under OA conditions. No differences between the viability of oysters with the silenced perlucin were detected; however, there were differences in shell development (deformities and length) and in CaCO_3_ biomineralization.

Two transfection methods were employed: (1) using sperm to deliver the probe into the egg during fertilization and (2) soaking 6 h old larvae in a high concentration of probes for 24 h. It was hypothesized that these methods would reduce deformities and mortality caused by harsher methods of transfection such as electroporation or chemically altering the egg composition through decapsulation. There was negligible mortality from either treatment (<10%) and larvae with negative DsiRNA control did not have significantly more deformities than larvae without DsiRNA. Both methods were successful at significantly reducing the expression of perlucin, but there were advantages and disadvantages to each approach (Table S2). A major difference between the two methods was the length of transfection prior to exposure to *p*CO_2_ treatments. Larvae from Experiment 1 were transferred from their transfection vessel to the *p*CO_2_ treatments within 4 h of fertilization, whereas larvae from Experiment 2 were not moved to *p*CO_2_ treatments until 30 h after fertilization. The larvae from Experiment 1 were significantly smaller and experimental groups had greater deformities. The larvae in Experiment 2 did not have such pronounced differences. A study in the Pacific oyster (*C. gigas*) showed that the calcification process during embryogenesis is impacted by low pH of seawater, and in that investigation, only 5% of *C. gigas* larvae from the high-*p*CO_2_ treatment developed into normal D-shaped larvae compared to 68% of control larvae [49]. Another study also in *C. gigas* demonstrated that within the first 21 h of development, larvae under OA stress failed or delayed formation of the prodissoconch I shell (PD1) [50]. This might explain why larvae from Experiment 1 were smaller and had more deformities. The longer transfection time for Experiment 2 missed this initial development period under OA conditions. For OA studies, Experiment 1 would be more suitable, as it allows for exposure to experimental conditions almost immediately after fertilization. However, a drawback of Experiment 1 was that to ensure eggs were not fertilized, adults were strip spawned, which provided a smaller initial number of larvae compared to natural spawning and only allowed for one sampling point, and the biomineralization assay did not have enough replicates for statistical analysis. Strip spawning of oysters results in fewer viable larvae compared to natural or induced spawning procedures [51]. However, our limited results from Experiment 1 did show a reduction in biomineralization in silenced oysters. Batch soaking of larvae for transfection rather than injecting individuals with DsiRNA is less likely to have all individuals with silenced perlucin. Survivors that were sampled at later times might have been the individuals that did not have perlucin successfully knocked down, and the earlier deaths might be the oysters with perlucin inhibition. A double transfection of barnacle at multiple development stages proved to be more effective than a single transfection [52]. This experiment could have benefitted from a second transfection step, which could have reduced the variability within treatments that was seen in this study.

In addition to confirming that perlucin was inhibited in the gene-silencing treatments, it was critical to validate that perlucin was differentially expressed between *p*CO_2_ treatments in this cohort of oysters. Perlucin was chosen because of previous work in which four-day-old eastern oyster larvae significantly over-expressed this gene under OA stress. Perlucin expression was tested on day three for Experiment 1 and day five for Experiment 2. While perlucin had greater expression at both time points, it was only significant at day five. Perlucin expression was monitored for the first seven days post-fertilization in *Panopea globosa* exposed to control and OA conditions. On days one, two, three, five, six, and seven, larvae had significantly higher expression of perlucin; however, at day four, perlucin expression was not significantly different between larvae from either *p*CO_2_ condition [22]. During larval development, there are transient morphological changes such as development from egg to embryo to trochophore to formation of PD1 shell. Each stage has a unique mRNA signature and could explain the differences in gene expression between days.

After confirming both over-expression and successful inhibition of perlucin, the next step was to perform different assays to see if there were measurable phenotypic differences between larvae with silenced perlucin under acidified conditions and larvae without silencing in acidified conditions. Knocking down perlucin did not impact viability in acidified or control conditions. In the time frame investigated (up to 5 days), perlucin does not appear to be linked to viability, but this does not indicate that larvae would have survived to metamorphosis. Studies have demonstrated that there are not always differences in mortality of larvae between *p*CO_2_ conditions within the first few days of exposure [53,54]. Exposure to high *p*CO_2_ can lead to significant impacts on larval development, such as thinner shells and smaller size, that are not followed by immediate mortality [55], but these negative impacts could increase vulnerability later on and reduce settlement and success of metamorphosis. If larvae were to survive, there could also be carry-over effects. A study in Olympia oyster (*Ostrea lurida*) demonstrated that effects from OA incurred during larval development were even more evident after settlement. Carry-over effects lasted 1.5 months after juveniles were transferred to control conditions [56]. Even if larvae did metamorphosize, thinner and deformed shells could make them more vulnerable to predation or environmental stressors.

While there were no reductions in viability following the silencing of perlucin, larval shell development, including both deformities and length, was impacted. Ninety-two percent of larvae with inhibited perlucin and in OA conditions were deformed. They were significantly more deformed than larvae from any other treatment. Larvae were considered deformed if there was lack of a shell, damaged shell valves, shell degradation, deterioration of the hinge, or abnormalities in the velum such as protrusion of the velum. This study did not use electron microscopy and so could have missed some additional deformities from OA, such as pitted shells or changes in shell microstructure [57,58,59]. These seriously deformed larvae would probably not survive through metamorphosis. Calcified structures of larvae are important for survival, including feeding, protection from predators, and buoyancy. Velum protrusions were a deformity observed in this study and the velum is the main swimming and feeding organ of larvae. OA studies have shown feeding is linked to resilience to OA [56,60,61] and a damaged velum could mean larvae are more susceptible to OA. This general trend of deformities was confirmed in Experiment 2; however, 28% rather than 92% of silenced larvae under OA conditions were deformed. Across the board there were much fewer deformities in Experiment 2. Experiment 2 larvae for this assay were sampled at a later point than Experiment 1, so very deformed larvae that were likely caught in the earlier sampling point for Experiment 1 could have already died off. Larvae under OA conditions with perlucin silencing were not significantly different from DsiRNA-NC5 pH = 7.3 larvae, but they were significantly smaller than larvae just in acidified seawater. As mentioned before, these larvae were not exposed to high *p*CO_2_ until later, which might be why there is less of an effect of acidification. Another reason for differences between Experiments 1 and 2 is that larvae in Experiment 1 had perlucin inhibited during fertilization, whereas Experiment 2 larvae developed for 6 h with perlucin. Perlucin is involved in the initial formation of the PD1 shell [62] and is expressed within the first 24 h of development [22,63] in bivalve larvae. Differences observed here might indicate that perlucin is in fact crucial during the first 6 h of development.

Deformed larvae observed in this investigation were smaller than normal D-shaped larvae, indicating a relationship between deformities and shell length. For Experiment 1, silenced larvae in both *p*CO_2_ conditions were significantly smaller than their controls. There were greater deformities in Experiment 1 and the most deformed larvae were the smallest. They also had exposure to OA during earlier development. As mentioned above, deformed larvae may have had a reduced ability to eat, which meant less energy available for growth. This trend was not seen in Experiment 2, and it is interesting that Experiment 2 also had fewer deformities. However, there were differences in the mineralization of larval shells in Experiment 2. A cost of sustaining growth could be thinner shells. In the clam *Laternula elliptica*, low pH did not impact larval body size or growth; however, it impacted shell quality, where seemingly normal larvae were actually impacted at the ultrastructural level [58].

The significant reduction in mineralization seen here most likely indicates that despite similar growth, shells of silenced larvae in acidified conditions are compromised. Cross-polarized light microscopy has been used successfully as a proxy for measuring biomineralization [59,64,65,66]. Cross-polarized light is double-refracted when passing through CaCO_3_. Larval shells with greater CaCO_3_ should double-refract more light and the intensity of birefringence can be used as a proxy for biomineralization [65]. Silenced larvae under OA conditions had the least amount of biomineralization. Overall, larvae from all the acidified treatments had less biomineralization than the ambient seawater (pH = 8.2) conditions. OA decreases saturation levels of CaCO_3_ minerals such as aragonite. Larval shells of eastern oysters are composed of aragonite, which is less stable and more soluble than other CaCO_3_ minerals, making it likely more susceptible to the effects of OA. The shell length of D-stage *C. gigas* was negatively impacted by aragonite undersaturation and there was a 57% decrease in the mass of shell accreted under aragonite undersaturation [67]. OA conditions in this study had aragonite undersaturation (Ω_aragonite_ = 0.4), and larvae in this condition with perlucin inhibition had significantly less mineralization than both controls. A decrease in mineralization from inhibited perlucin is not surprising, as perlucin is a biomineralization protein that functions in the nucleation of aragonite crystals. Dodenhof et al. (2014) found that perlucin splice variants differentially behave and can change how they modulate the crystallization of CaCO_3_ [68]. The initial investigation that allowed us to select perlucin as a candidate target for this work showed that this gene had a missense SNP enriched in survivors of OA. This variant could be linked to a difference in the function (or efficiency) of perlucin, with survivors of OA containing a variant that enhances perlucin’s ability to modulate CaCO_3_ crystallization under acidified conditions. Furthermore, Weiss et al. (2000) suggested that perlucin may serve to connect the chitin and aragonite layers of shells [27]. Chitin is known to be important for the development and functionality of larval shells [69] and Liu et al. (2020) suggested that seawater acidification might lead to a decrease in chitin content which results in a failure to form the shell in oysters [50]. In fact, previous studies have demonstrated that chitin binding genes are upregulated under OA stress [70], possibly to combat this. The role of perlucin in providing a connection between chitinous and aragonite layers might be another pathway important for tolerance to OA in oyster larvae.

In this study, larvae in OA conditions had less CaCO_3_ mineralization than controls and were smaller and had greater deformities, which supports the growing literature addressing the impacts of acidification on bivalve larvae [59,71,72,73]. However, here we demonstrated that the larvae in OA conditions perform even worse when perlucin is silenced, which strongly suggests that perlucin is involved in mitigating the impacts of OA in eastern oyster larvae. We also demonstrate two methods used on bivalve larvae to successfully reduce expression of a target gene. This is one of a few studies to take this further step to functionally validate results from large-scale ‘omics’ experiments targeting the identification of molecular features associated with resilience to OA. It also provides a functional analysis of the perlucin gene. One hundred and thirty-five genes are classified as perlucin or perlucin-like in the eastern oyster genome [25], without verification of their functions. The results presented here help to verify that this perlucin gene does function in shell formation and provides protection against OA.

## 4. Materials and Methods

### 4.1. Perlucin Gene Sequence Characterization

As discussed above, the choice of the target gene was based on our previous work in *C. virginica*. Briefly, a set of experiments targeting the characterization of oyster responses to OA allowed the identification of a perlucin gene that is upregulated (high-throughput transcriptomic profiling using RNASeq) in oyster larvae and juveniles under OA stress. To ensure the targeted perlucin was a good choice, it was aligned with other perlucins in the *C. virginica* genome (using the BLAST algorithm of the NCBI database and Clustal Omega) to ensure specificity and reduce cross-reactivity of our probes.

**Figure 8:**
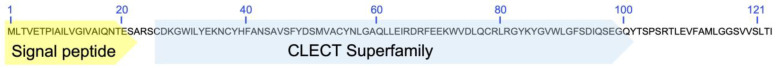
Protein sequence of the perlucin (*LOC111110737*). Signal peptide is in yellow and C-lectin domain is in blue. The length and number of amino acids are shown.

The target perlucin (*LOC111110737*; transcript XM_022447329.1; protein XP_022303037.1) gene is 366 nucleotides in length and encodes 121 amino acids (aa), including a signal peptide (22 aa) and a classical C-lectin domain (74 aa) long (Figure 8).

### 4.2. DsiRNA Synthesis

The nucleotide sequence of the candidate perlucin was uploaded to the DsiRNA design tool from IDT (Skokie, IL, USA; https://www.idtdna.com/site/order/designtool/index/DSIRNA_CUSTOM accessed on 1 April 2021), which allowed the customization of three DsiRNAs (Table 1). Candidate DsiRNAs were then compared against the *C. virginica* genome (GCF_002022765.2_C_virginica-3.0 from NCBI) to avoid cross-reactivity by having more than two mismatched bases in the first 19 bases on the plus strand. In addition to the DsiRNAs, a negative control (NC5) that did not target any part of the *C. virginica* genome was also synthesized [36]. This negative control serves to evaluate the unintended consequences (e.g., immune response) of introducing foreign RNA into oyster cells.

### 4.3. Animals

Seventy-seven ripe adult oysters (4–5 cm long) were collected from floating bags (at East Hampton Shellfish Hatchery, Montauk, NY, USA, on 2 June 2021). Oysters were scrubbed to remove epibionts and sediment and placed in a sea table with raw seawater (17 °C, 31 PSU, pH 7.84) and fed ad libitum with cultured algae (*Tetraselmis* spp., *Isochrysis galbana, Pavlova lutheri*, and *Chaetoceros muelleri*). Seawater was allowed to naturally increase to room temperature (~20 °C, reached within ~1 h). To stimulate spawning, oysters were submitted to a heat shock by adding hot seawater (27 °C) following thermal cycling recommendations of [74]. When oysters began spawning, males and females were removed from the sea table, rinsed with seawater and placed in individual aquaria. A subset of oysters were used for strip spawning, which is required for one of the two transfection methods tested (detailed below).

### 4.4. RNAi

Due to the lack of information regarding the transfer of genetic material into bivalve larvae, two transfection approaches were evaluated. The first approach used the sperm as a vehicle to carry the DsiRNA into the eggs, without any chemical agents or decapsulation (chemically removing the protective layer of eggs) during the fertilization process. The second approach relied on passive uptake of the DsiRNA using a previously successful larva soaking delivery method [35].

#### 4.4.1. Experiment 1

To ensure eggs were not fertilized before adding the DsiRNA, oysters were strip spawned [75]. Briefly, oysters were shucked and gonad material was scraped into 0.2 µm filtered seawater (FSW) and examined under a microscope to determine gamete type. Eggs and sperm were collected and remained separated. Three subsamples of pooled sperm (10 mL) were then incubated with one of the following: a cocktail of DsiRNA targeting perlucin (4 nmol), DsiRNA-NC5 (4 nmol), and no DsiRNA (seawater). After 15 min incubation, pooled eggs were added to each of the three groups of sperm. To allow for fertilization and transfection, eggs and sperm remained in their original vessels for 4 h before being moved to larvae culture flasks. Viability of developing larvae was assessed microscopically, after the transfection step and before the addition to *p*CO_2_ treatments, using ciliary movement to determine if the transfection method induced mortality. Larvae (~300,000 larvae/replicate) were then moved to acidified seawater (*n* = 3, detailed below) or control seawater (*n* = 3) for the resulting treatments: DsiRNA-perlucin/pH = 8.2; DsiRNA-perlucin/pH = 7.3, DsiRNA-NC5/pH = 8.2; DsiRNA-NC5/pH = 7.3; no DsiRNA/pH = 8.2; no DsiRNA/pH = 7.3 (Figures S2 and S3).

The number of embryos was low and just allowed the collection of one larvae sample after three days.

#### 4.4.2. Experiment 2

Sperm and eggs collected from oysters placed in individual aquaria were mixed to allow fertilization. This step was confirmed by the microscopic observations of polar bodies released by the embryos during meiosis. Six hours post-fertilization, the larvae were split into three batches that received one of the following: a cocktail of the DsiRNAs targeting perlucin (6 nmol into 4 L of 0.2 µm FSW), DsiRNA-NC5 (6 nmol into 4 L of FSW), and 4 L of FSW. The larvae remained in the solutions for 24 h with gentle bubbling of ambient air. Viability was assessed to determine if transfection caused mortality and then larvae (~300,000/replicate) were moved to either acidified (*n* = 3) or control (*n* = 3) seawater (Figures S3 and S4). Larvae were sampled at days three and five post-fertilization.

### 4.5. Carbonate Chemistry and Larvae Care

After transfection, larvae were moved to FSW bubbled with air only for ambient conditions (pH~8.2, *p*CO_2_~400 ppm) or 5% CO_2_ and air mixed via multi-gas channel proportioners (Cole Parmer; [76]) for acidified seawater (pH~7.3, ~3500 ppm) (Table S3). Aquaria were submerged in a water bath held at 25 °C (recommended for oyster larva development; [74]). Water changes were performed every other day and larvae were fed ad libitum with cultured *Isochrysis* sp. and *Pavlova lutheri* [74]. Samples for dissolved inorganic chemistry analysis were collected and read using a VINDTA 3D system, and certified reference material was analyzed during the beginning, middle, and end of runs for quality control (provided by Andrew Dickson, Scripps Institution of Oceanography). DIC, CO_3_, *p*CO_2_, alkalinity, and Ω_aragonite_, and Ω_calcite_ were calculated using the seacarb package in R, with known first and second dissociation constants of carbonic acid in seawater [77].

### 4.6. Real-Time PCR

Approximately 100,000 larvae (per replicate per *p*CO_2_ treatment per condition) were collected at day 3 for Experiment 1 and day 5 for Experiment 2, flash frozen, and stored at −80 °C for gene expression analyses. RNA was extracted using Monarch^®^ Total RNA Miniprep Kit (New England BioLabs, Ipswich, MA, USA) following manufacturer’s protocol. cDNA was synthesized from extracted mRNA using M-MLV reverse transcriptase (Promega, Madison, WI, USA) and used as a template with each set of primers (Table 1). Relative quantification of transcripts from each target gene was carried out in 10 μL reactions with Takyon Low Rox SYBR 2X MasterMix blue dTTP (Eurogentec, Seraing, Belgium), 100 nM final primer concentration, and 5 ng of RNA-equivalent cDNA. The PCR reactions were performed using QuantStudio 6 Flex Real-Time PCR System (Applied Biosystems, Waltham, MA, USA). Each run was followed by a melting curve program for quality control. PCR efficiency (E) was determined for each primer pair by determining the slopes of standard curves obtained from serial dilutions of cDNA (E = 103.98%). The correct amplification products were confirmed using gel electrophoresis. Perlucin expression levels were normalized to the 18S gene and relative transcript levels were calculated using the (2^−ΔΔCt^) method [78]. Real-time PCR data (ΔCt) were also analyzed using a one-way ANOVA and are presented as relative fold-change. Pairwise multiple comparisons were performed using the Student–Newman–Keuls method. A *t*-test was performed for relative expression of perlucin between acidified and control larvae.

### 4.7. Assessment of Larva Viability, Growth, Shell Development, and Biomineralization

Samples were collected to assess viability, growth, shell development, and biomineralization. Viability was assessed microscopically using ciliary movement. Larvae were sieved and rinsed into a graduated cylinder and a 0.5 mL sample was taken (in triplicate), and the average number of live and dead larvae was calculated [74]. The percent of live larvae was compared between treatments (*n* = 3 per treatment) using a 1-way ANOVA and post hoc comparison of least-square means. While checking for viability, larvae were classified as deformed or normal. The classification to distinguish between normal and abnormal shells was: absence of shell, damaged valves, shell degradation, presence of a convex hinge, and velum protuberances (examples of deformities in Figure S5). The percent of deformed larvae was compared between treatments (*n* = 3) using a 1-way ANOVA with post hoc comparison of least-square means. Shell lengths of 10 larvae per replicate were assessed using AmScope ISCapture (version 3.9.0.601) measuring tool by measuring the anterior-to-posterior length parallel to the hinge. Lengths were compared between treatments (*n* = 3) using a nested ANOVA (aquaria random effect; probe/*p*CO_2_ treatment fixed effect) with post hoc comparison of means with Tukey and Bonferroni correction. Growth rate was also determined and compared using a nested ANOVA with post hoc comparison of means using Tukey’s test. Larvae were analyzed for CaCO_3_ biomineralization using cross-polarized light microscopy [27,59,65,66]. Larval shells with higher CaCO_3_ refract more light and this can be used as a proxy to evaluate shell biomineralization. Birefringence was determined for 10 larvae per replicate (30 per *p*CO_2_/probe treatment) using Image J software (version 1.53; [79]). Images were converted to grayscale, and the mean of grayscale in pixels was determined for each larva (Figure S6) and assigned a birefringence index (BI). Biomineralization was evaluated for live larvae only, and differences were evaluated using nested-ANOVA and Tukey post hoc tests.

### 4.8. Statistical Analysis

All statistical analyses were performed in R version 3.6.3. Assumptions of normal distribution and homoscedasticity were determined using Shapiro–Wilk and Bartlett’s tests, respectively. Specific tests are described above. All results were deemed significant at *p* < 0.05.

## Figures and Tables

**Table 1:** Primers and DsiRNAs used in this study. The amplicon size of primers was 201 bp for 18S and 131 bp for perlucin.

Name	Sequence (5′–3′)	Tm
qRT-PCR primers		
18S-Forward	CGCCGGCGACGTATCTTTCAA	60.1
18S-Reverse	CTGATTCCCCGTTACCCGTTA	56.5
Perlucin-Forward	TTAGGTGGATCGGTGGTGAGC	59.0
Perlucin-Reverse	CATTCCACCCGTTAGAGGCTC	56.9
DsiRNAs		
DsiRNA-Perlucin 1 Sense	rArGrArUrArGrArCrArArUrArArCrUrGrArArUrArUrGrCAA	
DsiRNA-Perlucin 1 Antisense	rUrUrGrCrArUrArUrUrCrArGrUrUrArUrUrGrUrCrUrArUrCrUrGrC	
DsiRNA-Perlucin 2 Sense	rGrArArGrUrArUrUrCrGrCrUrArUrGrUrUrArGrGrUrGrGAT	
DsiRNA-Perlucin 2 Antisense	rArUrCrCrArCrCrUrArArCrArUrArGrCrGrArArUrArCrUrUrCrUrA	
DsiRNA-Perlucin 3 Sense	rCrGrGrGrUrGrGrArArUrGrArCrUrUrCrCrCrArUrGrUrUCA	
DsiRNA-Perlucin 3 Antisense	rUrGrArArCrArUrGrGrGrArArGrUrCrArUrUrCrCrArCrCrCrGrUrU	
DsiRNA-NC5 Sense	rCrArUrArUrUrGrCrGrCrGrUrArUrArGrUrCrGrCrGrUrUrArG	
DsiRNA-NC5 Antisense	rUrGrGrUrArUrArArCrGrCrGrCrArUrArUrCrArGrCrGrCrArArUrC	

## Data Availability

Not applicable.

## Supplementary Materials

The following supporting information can be downloaded at: https://www.mdpi.com/article/10.3390/ijms24043661/s1.
