# Supplementary material for: RNAi Silencing of the Biomineralization Gene Perlucin Impairs Oyster Ability to Cope with Ocean Acidification
Source: Int J Mol Sci. 2023 Feb 11;24(4):3661. doi: 10.3390/ijms24043661 (PMC9961701; doi:10.3390/ijms24043661)
Supplement: Supplementary file 1 [file ijms-24-03661-s001.zip › ijms-2085389-supplementary.pdf]

Table S1. Perlucin expression was compared between treatments for Experiment 1 (day 3) and Experiment 2 (day 5) and this table shows results of pairwise multiple comparison Student-Newman-Keuls test after ANOVA, n=3

**Comparison**	**P-value**
Experiment 1 Dsi to SW (control)	0.002
Experiment 1 Dsi to NC (control)	0.001
Experiment 1 NC to SW (control)	0.45
Experiment 1 Dsi to SW (acidified)	0.048
Experiment 1 Dsi to NC (acidified)	0.02
Experiment 1 NC to SW (acidified)	0.379
Experiment 2 Dsi to SW (control)	0.027
Experiment 2 Dsi to NC (control)	0.015
Experiment 2 NC to SW (control)	0.0993
Experiment 2 Dsi to SW (acidified)	0.022
Experiment 2 Dsi to NC (acidified)	0.024
Experiment 2 NC to SW (acidified)	0.593

Footnote: Dsi: DsiRNA-Perlucin, NC: DsiRNA-NC5, SW: seawater

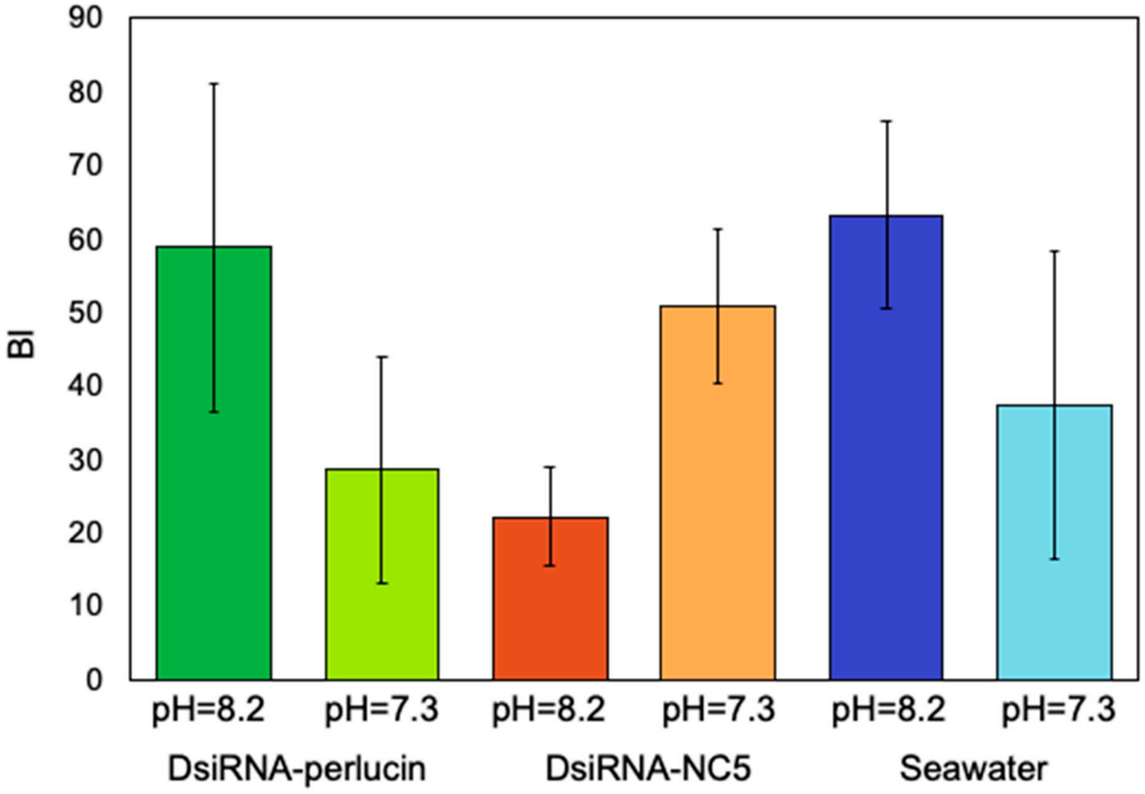

Figure S1. Shell birefringence index (BI, mean ± standard deviation) of oyster larvae from Experiment 1, day 3. N=30 individuals per treatment.

Table S2. Advantages and disadvantages to each transfection method

	Method 1	Method 2
Natural spawning	✗	✓
Time to pCO$_2$	✓	✗
Time to silence perlucin	✓	✗
Viability from transfection	✓	✓
Reduced perlucin expression	✓	✓
Multiple samples	✗	✓

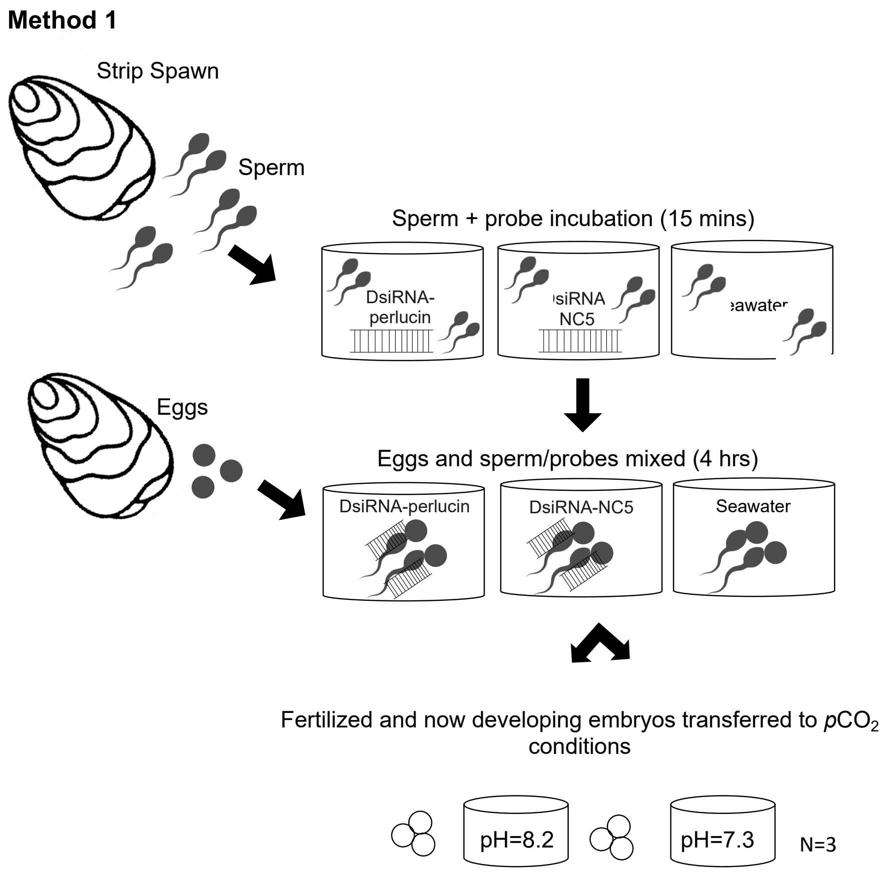

Figure S2. Experiment 1 design which used the sperm as a vehicle to carry the DsiRNA into the eggs.

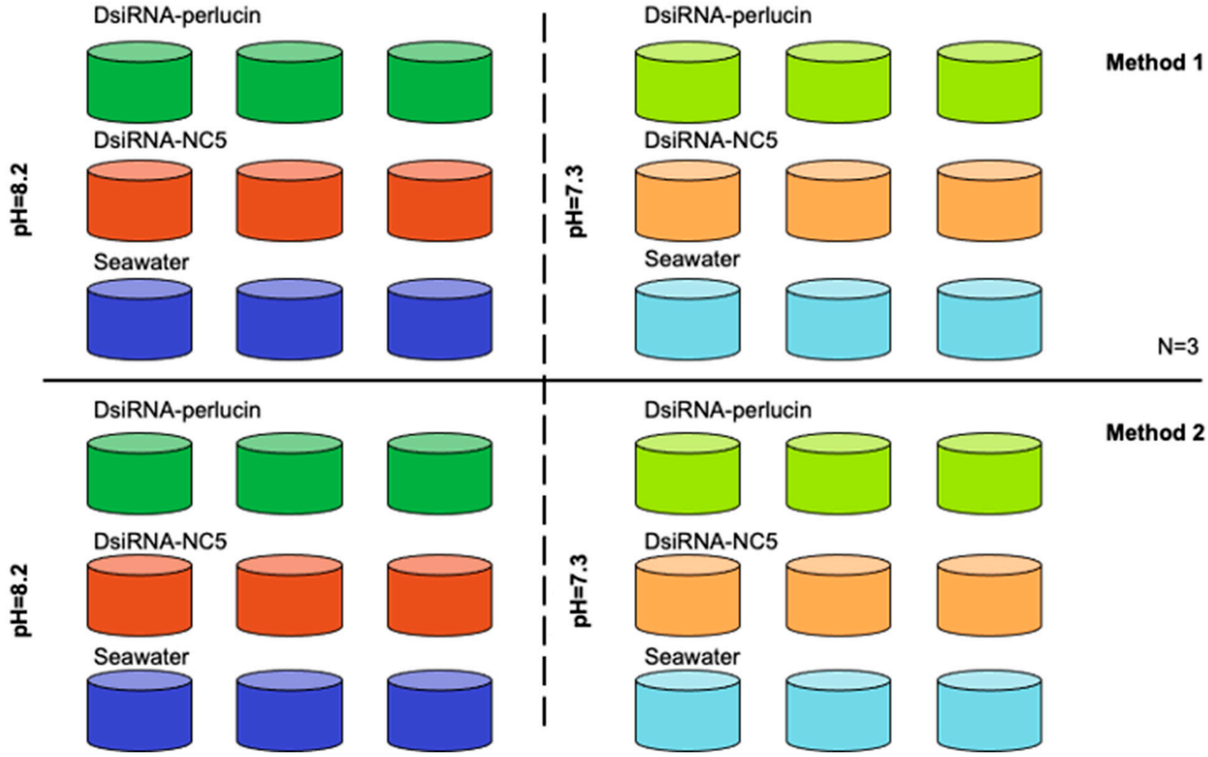

Figure S3. Experimental set up for $p\mathrm{CO_2}$ treatments

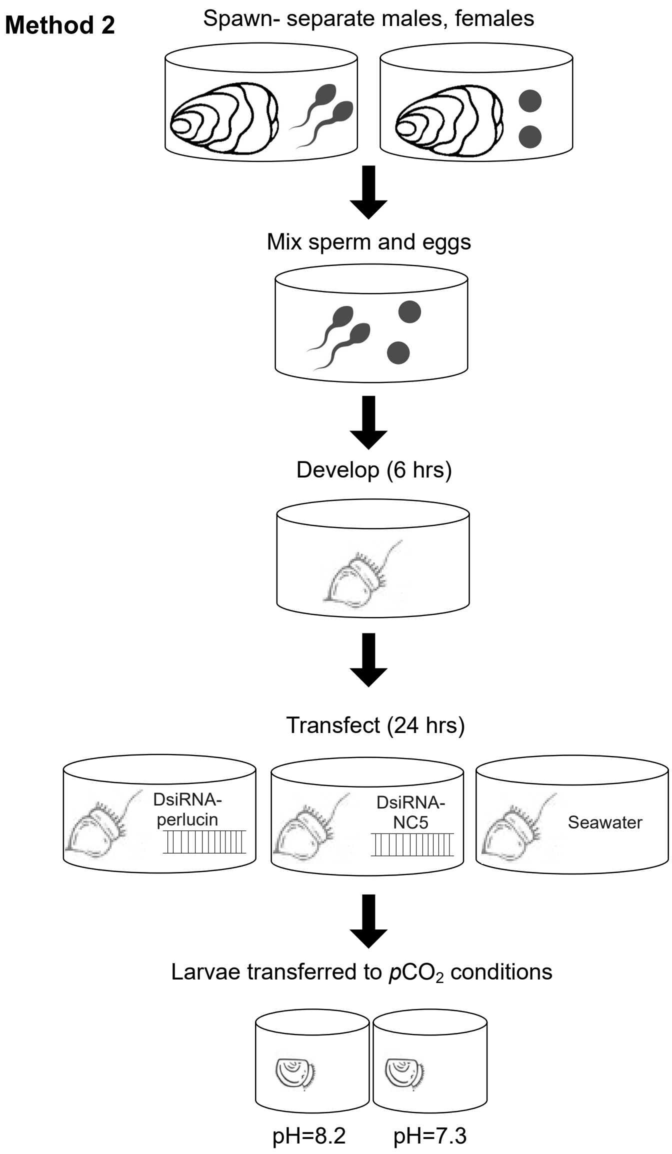

Figure S4. Experiment 2 design which relied on passive uptake of DsiRNA in developing larvae.

Table S3. Seawater carbonate chemistry values ± SD for gene silencing experiments. GS refers to DsiRNA-perlucin; NC refers to DsiRNA-NC5; SW refers to seawater.

	**pH_T**	**pCO_2** (uatm)	**Ω Ca**	**Ω Ar**	**TCO_2 (µmol L^{-1})**	**CO_3^{2-} (µmol L^{-1})**	**Total Alkalinity (µmol L^{-1})**
Experiment 1							
GS OA	7.3±0.04	341±338	0.6±1	0.4±0.04	2088±64	24±2	2018±65
GS Control	8.2±0.01	411±12	4.1±0.1	2.6±0.1	1927±35	158±5	2141±40
NC OA	7.3±0.02	3335±229	0.6±0.01	0.4±0.01	2081±80	24±0.4	2013±72
NC Control	8.2±0.00	407±4	4.2±0.03	2.7±0.02	1914±19	160±1	2131±19
SW OA	7.3±0.03	3508±151	0.6±0.1	0.4±0.04	2062±82	22±2	1987±87
SW Control	8.2±0.01	402±0.05	4.2±0.1	2.7±0.1	1909±38	161±3	2126±39
Experiment 2							
GS OA	7.3±0.01	3708±239	0.5±0.02	0.3±0.01	2017±97	20±1	1934±90
GS Control	8.2±0.00	386±2	4.1±0.02	2.6±0.01	1867±9	159±1	2084±10
NC OA	7.3±0.02	3571±120	0.6±0.04	0.4±0.02	2164±15	24±1	2088±121
NC Control	8.2±0.01	368±3	4.3±0.2	2.8±0.1	1891±32	160±1	2131±19
SW OA	7.3±0.1	3861±436	0.6±0.1	0.4±0.03	2149±33	22±2	2062±23
SW Control	8.2±0.02	380±11	4.4±0.2	2.8±0.1	1936±48	170±9	2167±58

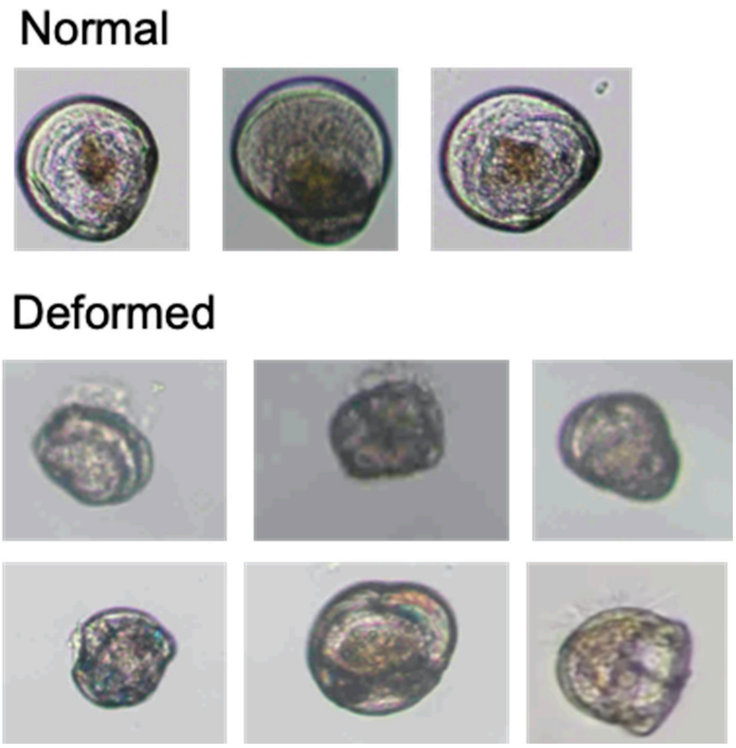

Figure S5. Representative photos of normal (top) and deformed (bottom) eastern oyster larvae from gene silencing experiments

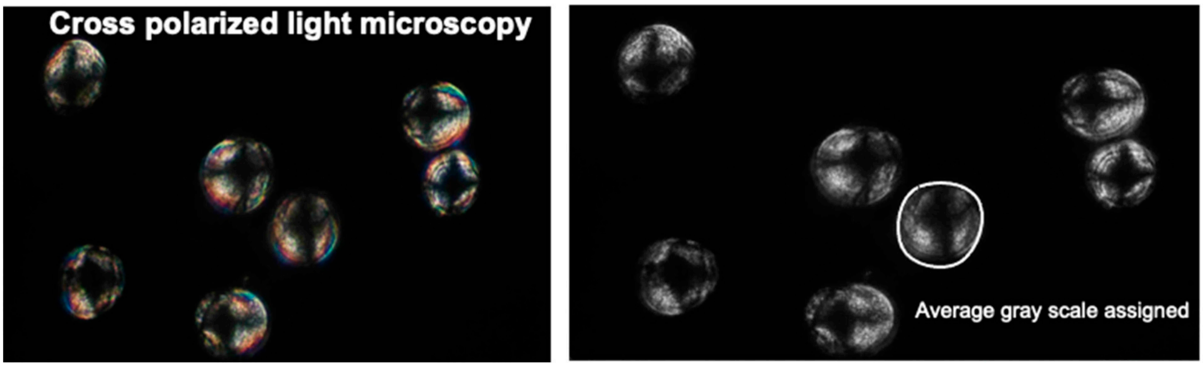

Figure S6. Photos were taken using cross polarized light microscopy to measure birefringence. Images were converted to gray scale, and individual larvae were circled and assigned a value ranging between 0 and 255 using ImageJ software.
